# Draft Genome Sequences of Novel *Campylobacter* Species Isolated from Nonhuman Primates

**DOI:** 10.1128/MRA.00146-20

**Published:** 2020-04-09

**Authors:** Anthony Mannion, Zeli Shen, Alex Sheh, James G. Fox

**Affiliations:** a Division of Comparative Medicine, Massachusetts Institute of Technology, Cambridge, Massachusetts, USA; Indiana University, Bloomington

## Abstract

*Campylobacter* species are being increasingly isolated and associated with disease in humans and animals. Here, we describe four draft genome sequences of *Campylobacter* species from nonhuman primates. These include Campylobacter troglodytis, isolated from wild chimpanzees, and two likely new *Campylobacter* species isolated from a lemur, common marmoset, and cotton-top tamarin.

## ANNOUNCEMENT

*Campylobacter* species are Gram-negative, microaerophilic bacteria that colonize the gastrointestinal and urogenital tracts of human and animal species (1). Infection by *Campylobacter* species, namely, C. jejuni, is implicated in gastroenteritis, septicemia, and in some cases Guillain-Barré syndrome. In humans, infection usually occurs after exposure to contaminated food, particularly chicken, and/or water. Domestic, wild, and captive animals, especially avian species, are significant reservoirs for *Campylobacter* species (1). Recently, the Centers for Disease Control and Prevention reported an outbreak of diarrheal illness due to C. jejuni infection transmitted after contact with puppies (2). As such, novel *Campylobacter* species are being increasingly identified from numerous animal species populations and require characterization of their pathogenic potential and zoonotic risk. Previously, our lab isolated *Campylobacter troglodytis* from the feces of a wild population of human-habituated asymptomatic chimpanzees (3). This organism has since been detected in the feces of infants prone to enteric infectious diseases living in developing countries (4). In this report, we describe the draft genome sequences for *Campylobacter troglodytis* and three novel campylobacters isolated from the feces of an asymptomatic captive lemur with a history of vomiting, a cotton-top tamarin with idiopathic inflammatory bowel disease, and an asymptomatic common marmoset.

Fecal samples were collected and then homogenized in freeze medium (20% glycerol in brucella broth). Fecal mixtures were passed through 0.45-μm syringe filters onto CVA plates or tryptic soy agar plates with 5% sheep blood (Remel Laboratories, Lenexa, KS). Plates were incubated at 37°C under microaerobic conditions (80:10:10 N_2_-CO_2_-H_2_) in a vented jar for 48 hours, and suspect campylobacter colonies were further passaged for 24 to 48 hours and incubated at 37°C and 42°C. Isolates were confirmed as campylobacters on the basis of colony morphology, Gram staining, biochemical reactions, and 16S rRNA sequencing. Campylobacter isolates grown on blood agar plates under microaerobic conditions for 48 to 72 hours at 37°C were collected using sterile cotton swabs into sterile phosphate-buffered saline (PBS) and then centrifuged to prepare bacterial pellets. Genomic DNA was isolated from bacterial pellets using the MasterPure complete DNA and RNA purification kit or Roche High Pure PCR product purification kit. DNA libraries were prepared using a NexteraXT or QIAseq FX DNA library kit for the Illumina MiSeq instrument (2 × 250-bp or 2 × 300-bp paired-end reads). Raw sequence reads were decontaminated of adapters and quality trimmed using BBDuk (5) for *de novo* contig assembly with SPAdes (version 3.10.0), hosted by PATRIC (accessed 1 February 2020) (6). Genome annotation was performed using Prokaryotic Genome Annotation Pipeline (PGAP) (version 4.11) (7). The assembly statistics for the draft genomes are described in Table 1.

**TABLE 1:** Genome summary statistics

Isolate name	Host	No. of contigs	*N*_50_ (bp)	Coverage (×)	Genome size (bp)	G+C content (%)	No. of proteins	No. of tRNAs	No. of rRNAs	No. of reads after quality control	GenBank accession no.	SRA accession no.	Sequencing information
*Campylobacter troglodytis* strain MIT 05-9149A	Asymptomatic wild chimpanzee (Pan troglodytes)	295	40,326	107.6	2,945,785	35.2	2,691	41	3	1,474,488	QHLI00000000	SRR10919684	Roche High Pure PCR product purification DNA extraction kit, QIAseq FX DNA library kit, Illumina MiSeq 2 × 300-bp paired-end reads
*Campylobacter* species MIT 99-7217	Captive cotton-top tamarin (Saguinus oedipus) with inflammatory bowel disease	37	182,074	167.5	1,789,167	34.0	1,789	45	4	1,419,300	QHLJ00000000	SRR10919685	Roche High Pure PCR product purification DNA extraction kit, QIAseq FX DNA library kit, Illumina MiSeq 2 × 300-bp paired-end reads
*Campylobacter* species MIT 12-5580	Lemur (Eulemur collaris) with history of vomiting	24	290,385	125.99	1,829,339	34.7	1,796	40	3	962,920	NXLK00000000	SRR10919683	MasterPure complete DNA and RNA purification kit, NextraXT library kit, Illumina MiSeq 2 × 250-bp paired-end reads
*Campylobacter* species MIT 19-121	Asymptomatic captive common marmoset (Callithrix jacchus)	27	185,094	342.1	1,887,695	34.7	1,875	40	3	3,328,860	JAADJR000000000	SRR10977364	Roche High Pure PCR product purification DNA extraction kit, QIAseq FX DNA library kit, Illumina MiSeq 2 × 300-bp paired-end reads

**FIG 1:**
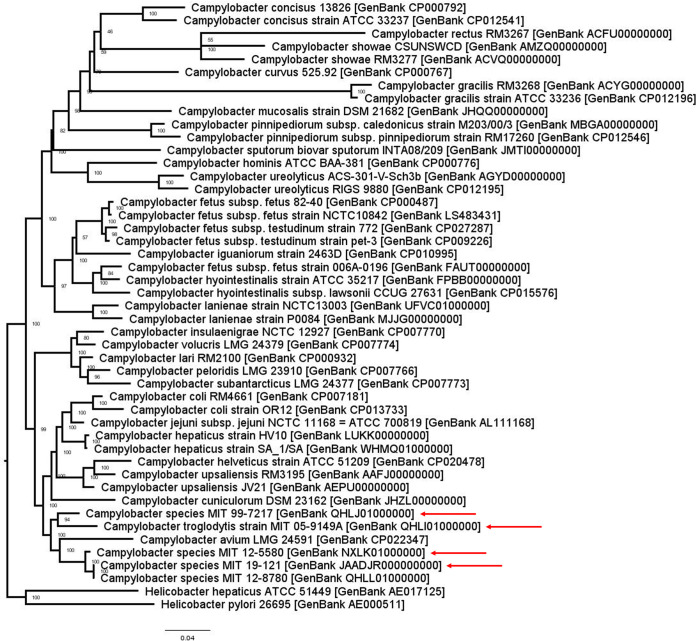
Pangenomic phylogenetic tree of representative genomes for each species in the *Campylobacter* genus. *C. troglodytis* strain MIT 05-9149A, *Campylobacter* species strain MIT 99-7217, *Campylobacter* species strain MIT 12-5580, and *Campylobacter* species strain MIT 19-121 (red arrows) are located in a distinct clade that also includes *Campylobacter* species strain MIT 12-8780 from the white-faced saki as well as *C. avium*.

A pangenomic phylogenetic tree created from the binary matrix of PATRIC global protein family groups with IQ-TREE (version 1.6.12) (8, 9) indicated that all novel genomes belonged to the *Campylobacter* genus but clustered within a distinct clade that includes C. avium (Fig. 1). Average nucleotide identity (ANI) and digital DNA-DNA hybridization (dDDH) analysis using pyani (version 0.2.10) (10) and the Genome-to-Genome Distance Calculator (version 2.1; accessed 3 April 2020) (11), respectively, confirmed that all the genomes were novel *Campylobacter* species. Also, ANI and dDDH analysis determined that *Campylobacter* species strain MIT 12-5580 from lemurs and *Campylobacter* species strain MIT 19-121 from marmosets are both the same species as *Campylobacter* species strain MIT 12-8780 from the white-faced saki (GenBank accession number QHLL01000000) (12).

The novel *Campylobacter* species genomes encode notable virulence factor genes, including flagella, campylobacter invasion antigen B (*ciaB*), and high-temperature requirement A serine protease (*htrA*) as determined through DIAMOND blast (version 0.9.29) (13) against the Virulence Factors Database (VFDB) (14). *Campylobacter* species strain MIT 99-7217 from a cotton-top tamarin, *Campylobacter* species MIT 12-5580 from a lemur, and *Campylobacter* species MIT 19-121 from a marmoset also harbor cytolethal distending toxin (CDT), a known genotoxin, which exacerbates gastrointestinal inflammation and carcinogenesis (15, 16). Due to the challenges presented for successful culturing of *Campylobacter* species and because biochemical profiles and 16S rRNA sequences cannot always differentiate these species, the detection and accurate identification of these campylobacters may be underrepresented (1). Nevertheless, emerging *Campylobacter* species typically found in animal reservoirs, such as C. upsaliensis, C. lari, C. hyointestinalis, and *C. troglodytis*, have pathogenic potential and are being increasingly associated with gastrointestinal illness in humans (4, 17, 18). Therefore, increased attention of the pathogenic potential and zoonotic risk of novel *Campylobacter* species is warranted.

### Data availability.

Genome sequences have been deposited in GenBank under the accession numbers QHLI00000000, QHLJ00000000, NXLK00000000, and JAADJR000000000. Sequencing reads have been deposited in SRA under the accession numbers SRR10919684, SRR10919685, SRR10919683, and SRR10977364.

## References

[1] Man SM 2011 The clinical importance of emerging *Campylobacter* species. Nat Rev Gastroenterol Hepatol 8:669–685. doi:10.1038/nrgastro.2011.191. 22025030

[2] CDC. 2019 Outbreak of multidrug-resistant Campylobacter infections linked to contact with pet store puppies. CDC, Atlanta, GA.

[3] Kaur T, Singh J, Huffman MA, Petrzelkova KJ, Taylor NS, Xu S, Dewhirst FE, Paster BJ, Debruyne L, Vandamme P, Fox JG 2011 *Campylobacter troglodytis* sp. nov., isolated from feces of human-habituated wild chimpanzees (Pan troglodytes schweinfurthii) in Tanzania. Appl Environ Microbiol 77:2366–2373. doi:10.1128/AEM.01840-09. 21278267 PMC3067447

[4] Platts-Mills JA, Liu J, Gratz J, Mduma E, Amour C, Swai N, Taniuchi M, Begum S, Penataro Yori P, Tilley DH, Lee G, Shen Z, Whary MT, Fox JG, McGrath M, Kosek M, Haque R, Houpt ER 2014 Detection of Campylobacter in stool and determination of significance by culture, enzyme immunoassay, and PCR in developing countries. J Clin Microbiol 52:1074–1080. doi:10.1128/JCM.02935-13. 24452175 PMC3993515

[5] Mannion A, Fabian N, Stair M, Dzink-Fox J, Carrasco SE, Buckley-Jordan E, Annamalai D, Fox JG 2019 Draft genome sequences of *Klebsiella pneumoniae* strains isolated from immunocompromised NOD-scid gamma research mice. Microbiol Resour Announc 8:e00869-19. doi:10.1128/MRA.00869-19. 31624163 PMC6797528

[6] Wattam AR, Davis JJ, Assaf R, Boisvert S, Brettin T, Bun C, Conrad N, Dietrich EM, Disz T, Gabbard JL, Gerdes S, Henry CS, Kenyon RW, Machi D, Mao C, Nordberg EK, Olsen GJ, Murphy-Olson DE, Olson R, Overbeek R, Parrello B, Pusch GD, Shukla M, Vonstein V, Warren A, Xia F, Yoo H, Stevens RL 2017 Improvements to PATRIC, the all-bacterial Bioinformatics Database and Analysis Resource Center. Nucleic Acids Res 45:D535–D542. doi:10.1093/nar/gkw1017. 27899627 PMC5210524

[7] Tatusova T, DiCuccio M, Badretdin A, Chetvernin V, Nawrocki EP, Zaslavsky L, Lomsadze A, Pruitt KD, Borodovsky M, Ostell J 2016 NCBI Prokaryotic Genome Annotation Pipeline. Nucleic Acids Res 44:6614–6624. doi:10.1093/nar/gkw569. 27342282 PMC5001611

[8] Nguyen L-T, Schmidt HA, von Haeseler A, Minh BQ 2015 IQ-TREE: a fast and effective stochastic algorithm for estimating maximum-likelihood phylogenies. Mol Biol Evol 32:268–274. doi:10.1093/molbev/msu300. 25371430 PMC4271533

[9] Chernomor O, von Haeseler A, Minh BQ 2016 Terrace aware data structure for phylogenomic inference from supermatrices. Syst Biol 65:997–1008. doi:10.1093/sysbio/syw037. 27121966 PMC5066062

[10] Pritchard L, Glover RH, Humphris S, Elphinstone JG, Toth IK 2016 Genomics and taxonomy in diagnostics for food security: soft-rotting enterobacterial plant pathogens. Anal Methods 8:12–24. doi:10.1039/C5AY02550H.

[11] Meier-Kolthoff JP, Auch AF, Klenk H-P, Göker M 2013 Genome sequence-based species delimitation with confidence intervals and improved distance functions. BMC Bioinformatics 14:60. doi:10.1186/1471-2105-14-60. 23432962 PMC3665452

[12] Clayton JB, Danzeisen JL, Johnson TJ, Trent AM, Hayer SS, Murphy T, Wuenschmann A, Elder M, Shen Z, Mannion A, Bryant E, Knights D, Fox JG 2019 Characterization of *Campylobacter jejuni*, *Campylobacter upsaliensis*, and a novel *Campylobacter* sp. in a captive non-human primate zoological collection. J Med Primatol 48:114–122. doi:10.1111/jmp.12393. 30536921 PMC6570501

[13] Buchfink B, Xie C, Huson DH 2015 Fast and sensitive protein alignment using DIAMOND. Nat Methods 12:59–60. doi:10.1038/nmeth.3176. 25402007

[14] Liu B, Zheng D, Jin Q, Chen L, Yang J 2019 VFDB 2019: a comparative pathogenomic platform with an interactive Web interface. Nucleic Acids Res 47:D687–D692. doi:10.1093/nar/gky1080. 30395255 PMC6324032

[15] He Z, Gharaibeh RZ, Newsome RC, Pope JL, Dougherty MW, Tomkovich S, Pons B, Mirey G, Vignard J, Hendrixson DR, Jobin C 2019 *Campylobacter jejuni* promotes colorectal tumorigenesis through the action of cytolethal distending toxin. Gut 68:289–300. doi:10.1136/gutjnl-2018-317200. 30377189 PMC6352414

[16] Fox JG, Rogers AB, Whary MT, Ge Z, Taylor NS, Xu S, Horwitz BH, Erdman SE 2004 Gastroenteritis in NF-kappaB-deficient mice is produced with wild-type *Camplyobacter jejuni* but not with *C. jejuni* lacking cytolethal distending toxin despite persistent colonization with both strains. Infect Immun 72:1116–1125. doi:10.1128/iai.72.2.1116-1125.2004. 14742559 PMC321575

[17] Kaakoush NO, Castano-Rodriguez N, Mitchell HM, Man SM 2015 Global epidemiology of Campylobacter infection. Clin Microbiol Rev 28:687–720. doi:10.1128/CMR.00006-15. 26062576 PMC4462680

[18] Francois R, Yori PP, Rouhani S, Siguas Salas M, Paredes Olortegui M, Rengifo Trigoso D, Pisanic N, Burga R, Meza R, Meza Sanchez G, Gregory MJ, Houpt ER, Platts-Mills JA, Kosek MN 2018 The other Campylobacters: not innocent bystanders in endemic diarrhea and dysentery in children in low-income settings. PLoS Negl Trop Dis 12:e0006200. doi:10.1371/journal.pntd.0006200. 29415075 PMC5819825

